# Differences in the Course, Diagnosis, and Treatment of Food Allergies Depending on Age—Comparison of Children and Adults

**DOI:** 10.3390/nu16091317

**Published:** 2024-04-27

**Authors:** Julia Kuźniar, Patrycja Kozubek, Krzysztof Gomułka

**Affiliations:** 1 Student Scientific Group of Internal Medicine and Allergology, Wroclaw Medical University, 50-369 Wroclaw, Poland; patrycja.kozubek@student.umw.edu.pl; 2 Department of Internal Medicine, Pneumology and Allergology, Wroclaw Medical University, 50-369 Wroclaw, Poland; krzysztof.gomulka@umed.wroc.pl

**Keywords:** food allergy, IgE, anaphylaxis, oral food challenge test, basophil activation test, skin prick test, oral immunotherapy, omalizumab, quality of life, non-IgE mediated food allergy, lipid transfer protein

## Abstract

Food allergy (FA) has become a common global public health issue, with a growing prevalence in the modern world and a significant impact on the lives of patients, their families, and caregivers. It affects every area of life and is associated with elevated costs. Food allergy is an adverse immune reaction that occurs in response to a given food. The symptoms vary from mild to severe and can lead to anaphylaxis. This is why it is important to focus on the factors influencing the occurrence of food allergies, specific diagnostic methods, effective therapies, and especially prevention. Recently, many guidelines have emphasized the impact of introducing specific foods into a child’s diet at an early age in order to prevent food allergies. Childhood allergies vary with age. In infants, the most common allergy is to cow’s milk. Later in life, peanut allergy is more frequently diagnosed. Numerous common childhood allergies can be outgrown by adulthood. Adults can also develop new IgE-mediated FA. The gold standard for diagnosis is the oral provocation test. Skin prick tests, specific IgE measurements, and component-resolved diagnostic techniques are helpful in the diagnosis. Multiple different approaches are being tried as possible treatments, such as immunotherapy or monoclonal antibodies. This article focuses on the prevention and quality of life of allergic patients. This article aims to systematize the latest knowledge and highlight the differences between food allergies in pediatric and adult populations.

## 1. Introduction

Food allergy (FA) is a pathological and potentially deadly immune reaction triggered by food protein antigens. Its prevalence is rising, and it is believed to affect up to 10% of people. The standard of care is not optimal; moreover, FA has a major negative impact on a patient’s life regardless of age. Allergic symptoms in adults differ from those in children, and adults are often allergic to foods less frequently seen in children. The diagnosis of food allergies relies on a combination of clinical and reaction history, skin and IgE testing, and oral food challenges. A significant amount of research has been directed at various forms of immunotherapy, including the oral, sublingual, and epicutaneous delivery routes. Moreover, food allergies have a negative impact on the quality of life due to the stress associated with eating allergenic foods. This makes the search for new preventive, diagnostic, and therapeutic solutions very important. The aim of this study is to provide an up-to-date review of the course of food allergies according to age. This article focuses on the factors contributing to prevention, symptoms, diagnosis, treatment, the occurrence of allergies in adults and children, and the impact on the quality of life of patients. The bibliographic research was performed in March 2024, and it was mostly limited to papers published in the last 6 years (there are only two articles from 2016 and two from 2017; the rest were published between 2018 and 2024). The articles were identified using the PubMed search, using key terms related to food allergies: “children”, “adults”, “differences between children and adults”, “IgE-mediated food allergy”, “epidemiology”, ”pathogenesis”, “prevention”, “diagnosis”, “treatment”, “quality of life”, “non-IgE mediated food allergy”, “lipid transfer protein”, “anaphylaxis”, “oral food challenge test”, “skin prick test”, and “oral immunotherapy”. To avoid excluding important studies, the research was not restricted by the type of publication or study design.

## 2. Children

### 2.1. General Information

Food allergy (FA) is an immune reaction caused by exposure to a specific food antigen. FA can take the form of IgE-mediated, non-IgE-mediated, or their combinations [1]. Non-immunoglobulin E-mediated gastrointestinal food allergic disorders (non-IgE-GI-FA) are being increasingly diagnosed in children. Three main disease entities can be distinguished: food protein enteropathy (FPE), food protein enteritis syndrome (FPIES), and food protein allergic proctitis (FPIAP) [2]. Other examples of non-IgE-mediated food allergies include coeliac disease and allergic proctocolitis. Mixed IgE- and non-IgE-mediated food allergies include atopic dermatitis and eosinophilic gastrointestinal disorders, such as eosinophilic esophagitis, eosinophilic gastritis, eosinophilic gastroenteritis, eosinophilic enteritis, and eosinophilic colitis [3]. The main differences between IgE-mediated and non-IgE-mediated food allergies are pathophysiology and the time from exposure to the allergen to the symptom’s manifestation [1]. IgE-mediated allergies belong to the first type of hypersensitivity and symptoms appear rapidly, usually within minutes to hours of exposure to the allergen [3,4].

Symptoms of IgE-mediated allergies affect many groups of organs and have varying degrees of severity. The most common symptoms in both children and adults include skin symptoms (rash, angioedema, and urticaria), respiratory symptoms (wheezing, sneezing, cough, itching, rhinitis, and stridor), digestive system symptoms (nausea, vomiting, diarrhea, and abdominal pain), cardiovascular symptoms (tachycardia and drop in blood pressure), neurological symptoms (mental status changes—confusion, anxiety, and loss of consciousness), and urinary symptoms (loss of bladder control) [3].

**Figure 1:**
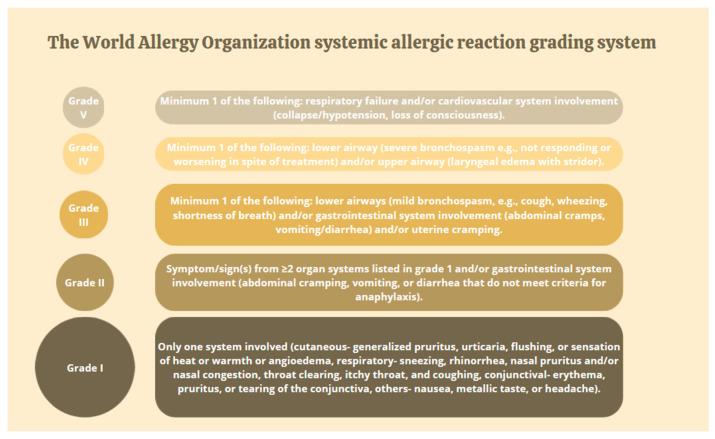
WAO systemic allergic reaction grading system [3].

The most serious allergic reaction in children and adults is anaphylaxis. According to the World Allergy Organization (WAO), systemic allergic reactions are divided into five grades. In this classification, grades 1 and 2 do not represent anaphylaxis. Some grades of 3 and all 4 and 5 grades are consistent with the definition of anaphylaxis. In pediatric patients, salivation and neurological symptoms should be taken into consideration. The classification is presented in Figure 1 [3]. Anaphylaxis is most often reported in children aged 0–4 years; however, fatal or near-fatal incidents occur later in life—in teenagers and adults up to 40 years. Regardless of age, the risk of severe disease is slightly higher in men [5].

The foods most likely to cause anaphylaxis vary with age. Anaphylaxis in children is most often caused by the consumption of hen’s eggs, cow’s milk, nuts, and wheat. In adults, however, its origin is of great importance. Among the inhabitants of Central Europe, anaphylaxis is most often caused by peanuts, tree nuts, seeds like sesame, wheat, and shellfish. In North America and Australia, anaphylaxis caused by peanut and tree nuts predominates; in Asia, the most common anaphylaxis is related to the consumption of shellfish; and in the Middle East, the greatest risk is associated with the consumption of sesame seeds [6]. In Europe and the USA, 6 to 8% of children suffer from food allergies. The number of patients has increased over the last two decades [1]. In the Japanese population, the number of children suffering from FA decreases from 10% in infancy to 4.5% in school children [7].

In theory, any protein can cause an allergic reaction [8]. Cow’s milk allergy is the most common FA in infancy; however, according to the opinion of the European Society of Pediatric Gastroenterology, Hepatology and Nutrition (ESPGHAN), cow’s milk allergy is overdiagnosed in exclusively breastfed, formula-fed, and both breastfed and formula-fed infants [9]. At the age of 1, the most common allergy is to hen’s eggs. From the age of 2 years, the most common allergy is peanut allergy. In school-age children, the most common FA includes peanut allergy, tree nut allergy, and fish and seafood allergy [10]. Allergies to chicken eggs, peanuts, soy, fish, tree nuts, shellfish, and sesame are common, and the prevalence of allergy varies depending on the population studied [11]. It is estimated that up to one-third of children with FA in the USA have multiple food allergies (a minimum of three different products induce symptoms) [12]. The occurrence of cross-reactivity is also important when test results show the presence of allergen-specific IgE toward specific food products in patients who do not experience clinical symptoms after exposure. A high degree of cross-reactivity occurs with shellfish, fish, tree nuts, and peanuts [13].

In the Mediterranean area, lipid transfer proteins (LTPs) are an important type of allergen. LTPs are present in plant foods. They have moderate to high molecular structure homology, which means that there is a risk of allergic reactions, even when consuming many different botanically unrelated substances. In the case of a lipid transfer protein allergy, most children report more than one trigger. Products containing LTP include various vegetables, fruits, tree nuts, seeds, and cereals. LTP allergy in children is significantly less known compared to adults [14]. The frequency of LTP allergies among children varies among different publications. In a study of 496 children assessed for multiple allergies living in the eastern Mediterranean region, 21% showed an allergy to LTP [15]. Among 281 patients presenting to a hospital in Portugal with suspected food allergies, 9% were diagnosed with an allergy to LTPs. In children, the most common reaction was to fruit (most often to peach) and tree nuts. Most children had their first symptoms before the age of 12. The most frequently reported symptom was urticaria. Compared to adults, there was no relationship between a higher level of IgE for Pru p 3 and systemic reactions to fruits from the Rosacea family. The risk of a reaction when consuming previously safe products later in life is significant. The main method of therapy is to avoid foods that can cause symptoms. However, patients should continue to eat tolerated foods without the peels, as they contain the most LTPs [14].

### 2.2. Risk Factors

The primary risk factor for food allergies is genetic predisposition [10]. A strong risk factor is a family history of atopy. The risk is increased by 40% in the case of one immediate family member with FA and by 80% in the case of two patients. Additionally, food sensitivity is observed in 66.6% of children and clinical reactions in 13.6% of children whose siblings suffer from food allergies [4].

Intestinal dysbiosis is also associated with an increased risk of food allergies. Some intestinal microflora profiles have a negative impact on the maturation and regulation of the immune system, which is associated with inflammation and allergic sensitization of the child’s intestine [10].

Obesity is a risk factor, according to some studies, which mainly affects female children [16]. There is a strong association between infant atopic dermatitis and food allergies. Recent studies have suggested that the presence of an inflammatory environment at the interface of the epithelium is a key factor in the development of allergic sensitization [10]. In an allergic march, food allergies occur at an early stage [7].

Vitamin D deficiency in pregnant women and children increases the incidence of food allergies. The risk of an allergic event in children with vitamin D deficiency increases by 68%. This relationship is most evident in the second year of life when an allergic episode is four times more likely to occur, and the likelihood of food sensitization increases by 56% [17].

Food allergies to products such as wheat, cow’s milk, soy, and eggs often develop in infancy and disappear during childhood. However, in the case of allergies to fish, peanuts, or shellfish, such a pattern is rare [10].

### 2.3. Diagnostics

In the first stage of diagnosis, it is important to collect a detailed interview, including, the suspicious food product and its intake by the child, the age at which symptoms first appeared and their repeatability, the nature of the symptoms, and the current exposure to the product [4,7,8,18].

Possible diagnostic options in children include:

Detection of specific IgE antibodies in blood: It should be added, however, that a positive test result does not always correlate with clinical symptoms. A high correlation index is noted for milk and eggs, and a lower one for soy [19]. Specific IgE towards certain allergen components has been shown to have higher specificity compared to specific IgE towards allergen extracts; therefore, they can be used to confirm allergies in the case of a suggestive medical history [20].

Skin Prick Test (SPT) enabling the differentiation of allergy-causing substances: In the case of unstable allergens, such as fresh fruits and vegetables, the prick–prick form is used. During the prick–prick test, the skin is punctured with a needle that was previously used to puncture the suspected product [7]. Sensitivity, specificity, positive predictive value, and negative predictive value for various allergens vary depending on many factors, including age, allergy prevalence in a given population, and cut-off values. For example, the values of the above-mentioned indicators can be compared for hen’s egg and soy allergies. In the case of a hen’s egg allergy test using a raw or partially cooked egg in a group of children under 2 years of age with a wheal size of 5 mm, the sensitivity was 4.2% and the specificity was 100% (positive predictive value = 100%); in the group of children under 14 years of age, for a 3 mm wheal, the sensitivity was 93% and specificity was 54–59% (positive predictive value = 79–80%); in the group of children under 18 years of age, for a 9 mm wheal, the sensitivity was 22% and specificity was 98.2% (positive predictive value = 95.6%). However, in the case of soy allergy in the group of children under 14 years of age, for a 3 mm wheal, the sensitivity was 21–29% and specificity was 85–88% (positive predictive value = 29–33%); in the group of children under 18 years of age, for a wheal size of was 3 mm, the sensitivity was 76–100% and specificity was 47–72% (positive predictive value = 30–35%). Greater diagnostic difficulties are noted in the case of soy allergies [19].

Basophil activation test (BAT) is a quantitative determination of IgE-dependent basophilic activation [7]. It is suggested to use BAT in cases of uncertain diagnosis of IgE-related food allergies to nuts or sesame in order to support the diagnosis [20].

The oral food challenge test best indicates the substance responsible for an allergic reaction, but it carries the risk of a severe adverse reaction. We distinguished different forms of the test: open test (the examiner and the examined patient know the composition of the challenge food), single-blind food challenge (the examined patient does not know the composition of the food), and double-blind placebo-controlled food challenge test—DBPCFC (both the examiner and the examined patient do not know the food composition) [7]. The double-blind placebo-controlled food challenge is the gold standard for diagnosing food allergies [1]. Immediate reactions most often occur 1–2 h after eating food, but they may occur even 24 h after consumption [7].

The diagnosis of non-IgE-mediated food allergies is much more difficult than that of IgE-mediated allergies. Currently, there are no approved tests for this. It is crucial to collect an extensive history and exclude IgE-mediated allergies. It is also possible to exclude the suspected product from the diet for a period of two to six weeks and then reintroduce it into the diet and observe the reaction [11].

To standardize the diagnosis of various forms of non-IgE-mediated food allergies, initial diagnostic criteria were created. In the case of acute FPIES, the major criteria include vomiting 1–4 h after ingestion of the suspected food and exclusion of IgE-mediated allergy symptoms. It is also very important that symptoms disappear after the removal of harmful food from the diet. Performing OFC may be beneficial. In the case of chronic FPIES, the pathognomonic feature helpful for diagnosis is the rapid disappearance of symptoms after withdrawal of the suspected food and their acute return after re-exposure. OFC is mandatory for the diagnosis of chronic FPIES. In laboratory tests of acute and chronic FPIES, it is possible to detect anemia, hypoalbuminemia, and thrombocytosis. When it comes to the diagnosis of FPE in children under 9 months of age, the presence of a characteristic clinical picture with vomiting and symptoms of malabsorption in the intestines is crucial. It is also helpful to perform a histopathological examination of an intestinal specimen. In the case of FPIAP, the main symptom of which is rectal bleeding, it is extremely important to exclude other causes of hematochezia. An attempt has also been made to eliminate suspicious foods with subsequent exposure and to monitor for the return of symptoms. Research is underway to introduce ultrasound examination for the diagnosis of non-IgE-mediated food allergies, as it often shows thickening of the walls of the small intestine and poor peristalsis. These symptoms disappear after eliminating the harmful food [21].

### 2.4. Treatment

Food allergy therapy can be divided into “passive” and “active” forms [1]. Passive therapy involves avoiding exposure to foods that trigger a reaction, which is still the main form of coping with the disease these days [1,21]. In the case of cow’s milk allergy in infants, the first-choice option is breastmilk, and the second-choice option is extensively hydrolyzed formula [22].

For breastfed infants, in some cases, a maternal exclusion diet may produce positive results. Some proteins, such as peanuts, when present in the mother’s milk, may cause an allergic reaction in a child [23]. Active therapies include specific allergen immunotherapy, including oral immunotherapy (OIT), sublingual immunotherapy (SLIT), and epicutaneous immunotherapy (EPIT). The goal of immunotherapy is to achieve “a state of tolerance” [1]. Studies on AR101 peanut immunotherapy have shown that the treatment is effective in increasing the number of peanuts a child can tolerate [24]. The new guidelines strongly recommend the use of peanut oral immunotherapy in some cases for children over 4 years of age. It is suggested to use peanut epicutaneous immunotherapy in children aged 4 to 11 years and oral allergen immunotherapy in children over 4 years of age in the case of severe IgE-mediated hen’s egg or cow’s milk allergy. Immunotherapy must be performed under close, specialized supervision using standardized, evidence-based protocols. The important fact is that, in children, the immune system is still developing and malleable, which means that many food allergies resolve before reaching adulthood. This is related to the controversy of using ICU at a young age versus waiting for the spontaneous resolution of symptoms [25]. It is suggested that children with food allergies should be reassessed at regular intervals to identify the possible development of spontaneous tolerance. Currently, increasing attention is focused on the use of biologics in the treatment of IgE-mediated FA in children, either as monotherapy or in combination with ICU. At present, in pediatric patients, one biologic drug is used, Omalizumab, which is a monoclonal antibody targeting IgE [26,27].

If an anaphylactic reaction occurs, a life-saving treatment involves the administration of adrenaline. Prehospital administration of adrenaline in young children may result in its reduced use in the Emergency Department but is also associated with a more frequent admission of the child for hospital observation [28].

In the case of non-IgE-GI-FA therapy, the most important thing is to remove the food causing the symptoms from the diet. In most cases, individual foods are removed sequentially without significant dietary restrictions. However, in the case of severe disease with dehydration and delayed normal development, significant restriction of food intake is required, with the gradual introduction of new foods into the diet and observation of the recurrence of symptoms. Cow’s milk is the most common cause of FPIES, FPE, and FPIAP. Nutritional therapy in this case involves replacement with an extensively hydrolyzed formula and, in 10–20% of cases with persistent symptoms, with an amino acid-based formula. Partially hydrolyzed formulas should be avoided in all cases [2].

### 2.5. Prevention

In the prevention of allergies, it is important to understand the mechanisms of development. There are three main hypotheses for the development of food allergies: the hygiene hypothesis (the occurrence of allergy in small families and in people not exposed to animals), the vitamin D hypothesis (the occurrence of allergy preceded by avoiding the sun and lack of exposure to UV radiation), and the dual-allergen exposure hypothesis (the occurrence of allergies after exposure to strong detergents) [1].

It is important to introduce potentially allergic foods into a child’s diet early [1,29]. According to the latest guidelines, allergenic foods, such as peanuts and boiled hen’s egg, should be introduced into the infant’s diet around 6 months of age, but not earlier than 4 months of age [30]. Some dietary recommendations in the perinatal and infancy periods that reduce the risk of food allergy include the following:

- Nuts: Various species of nuts should be introduced into the diet in an age-appropriate order in the first year of life, as an addition to breast milk feeding [31]. A meta-analysis of 3796 children showed with high certainty that introducing peanuts into a child’s diet between 3 and 10 months of age reduces the risk of food allergies [32]. Emerging evidence suggests that maternal peanut consumption has a protective effect during breastfeeding [33].
- Cow’s milk: In preventing cow’s milk allergy, it could be beneficial to use formulas rich in probiotics, prebiotics (including HMO—“analogs”), and synbiotics, as their beneficial effect on the composition of the gastrointestinal microflora has been proven, and an immunomodulatory effect is suggested [22].
- Chicken egg: The use of boiled, unpasteurized chicken eggs as complementary nutrition in infants reduces the incidence of egg allergy in childhood [34].

Activities such as the use of oral immunotherapy or hydrolyzed formulas to prevent cow’s milk allergy, and vitamin supplementation or fish oil supplementation is not effective in the prevention of food allergies in children [31]. It is not recommended to avoid foods considered allergic by the mother during pregnancy and lactation [35].

It is also important to emphasize the importance of breastfeeding, which does not reduce the risk of food allergies but has many benefits for the child [31]. Previous reports on the possible induction of an allergic reaction by proteins contained in the mother’s milk are currently not confirmed by research [36].

### 2.6. Quality of Life

Health-related quality of life (HRQL) in children and adolescents with food allergy is reduced, mainly noticeable in older children and children with severe manifestations of the disease. The fear of exposure to allergen outside the home and social consequences contribute to the reduction of HRQL. Allergies are also associated with higher levels of mental stress [37]. FA may be accompanied by other diseases that make it difficult for children to function in everyday life, for example, a strong relationship has been shown between the occurrence of FA and ADHD [38]. According to research, 20% of children with food allergies are exposed to bullying. It may take the form of verbal threats, touching with an allergen, or intentional cross-contact of their food with an allergen. Bullying can lead to sadness, depression, feelings of humiliation or shame [39]. A food allergy in a child affects the functioning of the entire family. There is a phenomenon of FA-specific anxiety (FAA), which also affects parents of sick children. FAA is associated with impaired family functioning and affects the parent’s feeling of anxiety [40]. When a child is diagnosed with a food allergy, parents may feel guilty, anxious and sad [41].

Additionally, children with food allergy are exposed to a slower growth rate, height is the most affected, and various nutritional deficiencies; in the case of cow’s milk allergy, the high risk of calcium, iodine and vitamin D deficiency is high [12,42]. In children with food allergy, allergy, there is an increased energy demand [12]. In order to prevent the negative effects of allergies, strong emphasis should be placed on an appropriate elimination diet, complementary preparations, and vitamin and mineral supplementation [42].

It is important to provide children at various levels of education with access to meals that do not contain specific allergens. Such action may be difficult in large centers such as nurseries, kindergartens and schools compared to home conditions [7].

Educating children and parents about the content of allergens in various food products has a beneficial effect on the course of FA. In many parts of the world, precautionary allergen labeling (PAL) appears on product packaging, which can help you make informed purchases [43].

It is also worth mentioning the occurrence of risky behaviors among teenagers and young adults, such as refusing to carry emergency medications or intentionally consuming products with a risk of reactions [5].

## 3. Adults

### 3.1. General Information

It is estimated that over 220 million people worldwide, including adults, suffer from food allergies. However, making precise calculations is difficult due to problems in objective diagnosis, the complexity of diagnostic tools, and, above all, the multitude and variable severity of clinical symptoms [44]. Food allergies are a major public health issue with an increasing number of patients, affecting up to 10% of adults. Allergic reactions to food can manifest in many different ways—from mild skin itching, abdominal pain, and rash to severe anaphylaxis or eosinophilic esophagitis. Food allergy is part of a wider group of clinical entities designated as ‘food hypersensitivity’, which includes any adverse reactions to food. If this reaction is immune-mediated, it is a food allergy; if it is not immune-mediated, it is food intolerance [20]. Food allergies are classified into IgE-mediated, non-IgE-mediated, and mixed pathogenesis. Specifically, it can be classified depending on whether the underlying mechanism is type I hypersensitivity (IgE-mediated), type III or type IV hypersensitivity (non-IgE-mediated), or a combination of IgE and cellular mechanisms (mixed) [20,45,46].

Adults may retain their childhood allergies into adulthood and develop novel, adult-onset allergies to previously tolerated foods. Recent data suggest that allergies that develop during childhood may be more likely to persist into adulthood than was previously assumed. Adult-onset allergies seem increasingly common. In total, 15–21% of patients with clinically confirmed FAs had adult-onset allergy and 48% of adults with FA reported at least one adult-onset FA. The specific allergy with the highest rate of adult-onset allergies is shellfish [47].

The allergens that cause reactions in adults are different from those in children. In adulthood, the most common allergens are shellfish (2.9%), milk (1.9%), peanuts (1.8%), other nuts (1.2%), and fish (0.9%). An increasing number of cases of primary allergies in adults associated with allergens are considered typical of children (eggs, soy, and wheat). Women over the age of 18 are more likely to suffer from the disease than men—the opposite of what is typical in childhood [45,48].

It is estimated that the first reaction occurs around the age of 30 [49]. Adolescents and young adults with food allergies are at an increased risk of severe reactions in comparison to children [26]. Symptoms usually appear several minutes to two hours after eating food [50].

Allergy to fruits and vegetables is most often observed in adults. Lipid transfer protein (LTP) allergy involves sensitization to LTP proteins, which are in plant foods; they are stable to heat, and digestion and play an important role in protecting plants from stressors such as drought and other negative environmental factors. The peach LTP allergen Pru p 3 is a prototypic marker of LTP sensitization, but there are other LTPs such as Mal d 3 (apple), Cit r 3 (orange), Bra o 3 (cabbage), Sin a 3 (mustard), Jug r 3 (walnut), and Cas s 8 (chestnut). Their IgE-binding capacity was first demonstrated in peach fruits and Parietaria pollens and was then detected by IgE immunoblotting in peach, cherry, apricot, and plum. However, there is an increasing number of different products containing LTP, for example, some pollens, particularly plane tree and mugwort, have been proposed as contributing to sensitization to food. Lipid transfer proteins can elicit systemic reactions in sensitized subjects. According to the data published in different countries, Pru p 3 is also a marker allergen for adult-onset symptoms. Patients suffering from an ‘nsLTP syndrome’ frequently display allergy manifestations with multiple plant-derived foods due to their ubiquitous distribution. Sensitization may occur through cutaneous exposure, via the gastrointestinal tract and inhalation, with cross-reaction symptoms. LTP allergy also significantly affects the quality of life, possibly due to the number of potential food triggers and the link to co-factors, making it difficult to predict whether a reaction to a particular food might occur [51,52]. Allergies to orally consumed forms of cannabis in adults may become an increasingly relevant public health problem because of its accessibility. Data indicate that cross-binding between nonspecific lipid transfer proteins from cannabis in patients sensitized to Can s 3 may result in clinical allergy symptoms to an array of fruits, vegetables, cereals, wine, beer, and tobacco. Alcoholic beverages are a rare allergy among adults. Recent data suggest that sensitization to nonspecific lipid transfer proteins from cereals causes reactions to beer. Moreover, alcohol is a well-known allergic cofactor of anaphylaxis. Alcohol increases intestinal permeability, which can lead to accelerated antigen uptake from the gastrointestinal tract into the bloodstream [53].

### 3.2. Risk Factors

Research shows that the most prevalent allergens, such as shellfish and nuts, are not consumed regularly, which results in loss of exposure to these allergens and hypersensitivity to them. In adults, the so-called PFAS—primary allergy to inhalant allergens—causes cross-reactions to food. For example, exposure to plant pollen may cause a food allergy to soy due to the similarity in the protein structure. The skin has also been proven to be a sensitizing pathway for food allergies following tick bites, as well as for milk, cheese, wheat, and soy allergies in adults using skin care products containing these ingredients. Moreover, the use of antacids contributes to the development of food allergies [48,54].

In addition, vitamin D deficiency, dysbiosis, and reduced consumption of omega-3 polyunsaturated acids also influence the occurrence of allergies. An increased level of hygiene in the population impairs the natural human microbiome [46].

People suffering from atopic dermatitis are predisposed to IgE-related allergies. Atopic dermatitis usually precedes the manifestation of food allergy symptoms. A disturbed skin barrier causes easier penetration of foreign antigens and activation of immune cells and additionally increases the level of IgE (atopy) [55].

Obesity, as a state of constant inflammation, also increases the risk of food allergies. Adipocyte hypertrophy causes the activation of dormant inflammatory cells in adipose tissue and the production of cytokines. In lean people, these cells are inhibited by Treg lymphocytes compared to those in obese patients. Pro-inflammatory immune factors enter the bloodstream and reach the intestines. Some studies are controversial because they do not link increases in BMI in adults with food allergies [56].

Moreover, climate change and environmental pollution are associated with an increased incidence of allergies in people, including adults [54]. Air pollution causes inhalation, skin, and food allergies. Climate warming leads to the movement of rodents and other pests to urban areas, which has an impact on the occurrence of allergies in the population. Higher temperatures and carbon dioxide increase the production of plant pollen, which has a cross effect on food allergies [57].

Professions also influence the risk of developing an allergy. People working as cooks, bakers, hairdressers, housewives, and medical workers are particularly at risk. Food proteins are often used as ingredients in skin and hair care cosmetics. Cooks and bakers have direct contact with food—grains, fish, and crustaceans. People working as medical professionals use hand disinfection or latex gloves (latex-fruit syndrome may occur after eating, among others, bananas, and avocados). These professionals are in contact with large amounts of allergens, which are skin irritants and cause cross-reactions with food allergens [58].

**Figure 2:**
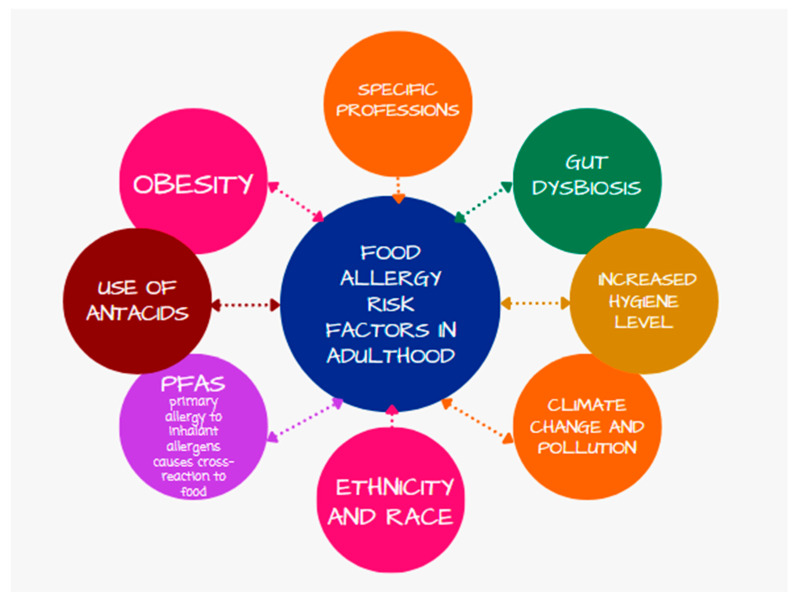
Summary of the food allergy risk factors in adulthood.

Research shows that ethnicity and race also influence the occurrence of food allergies. The NHANES analysis showed that the prevalence of food allergies among black people was 27%, while 13.8% among white people and 21.2% among Latin-origin patients [47]. A summary of the food allergy risk factors in adulthood is presented in Figure 2.

### 3.3. Diagnostics

Diagnosing food allergies in adults is a complex process. The most important step is the interview regarding current symptoms and the patient’s clinical history—symptoms indicating atopy in the past, especially in childhood, as well as symptoms of atopic dermatitis, allergic rhinitis, bronchial asthma, age at symptom onset, duration, treatment for previous reactions, suspected foods and their quantity and processing, route of exposure, co-factors of the reaction, settings, current food elimination, family history of atopic reactions, other allergies, and specific allergy tests cannot be interpreted in isolation from these data. The clinical history allows the identification of the possible mechanism of food allergies (i.e., IgE- or non-IgE-mediated) and the foods/allergens to be tested [20].

Diagnostics include physical examination, a double-blind placebo-controlled food challenge test (OFC test—oral food challenge), skin prick tests (SPT) used in the diagnosis of skin and food allergies, and blood serum allergy panels (allergen-specific IgE—SIgE) [46,59]. Currently, a promising method is molecular (component) allergy diagnostics (CRD—component resolved diagnostics). It enables the analysis of specific antibodies to specific molecules that are part of the allergen. In addition, research is ongoing on the measurement of specific IgE for antigen epitopes using microarrays. CRD is used in clinical practice to improve diagnostic accuracies and distinguish true allergic sensitization from cross-allergy [44,46]. A new method is the basophil activation test. BAT has higher specificity than traditional tests and allows the prediction of a severe allergic reaction during OFC [46,59]. BAT can be used to confirm allergy diagnoses by SIgE or SPT measurement tests. However, the diagnostic benefits of using BAT may vary among allergens [60]. An upcoming diagnostic method is the mast cell activation test (MAT), which, compared to BAT, uses plasma rather than fresh whole blood, but further work is needed on the standardization of procedures and laboratory standards as well as financial viability before these procedures become suitable for routine clinical diagnostics [61].

**Figure 3:**
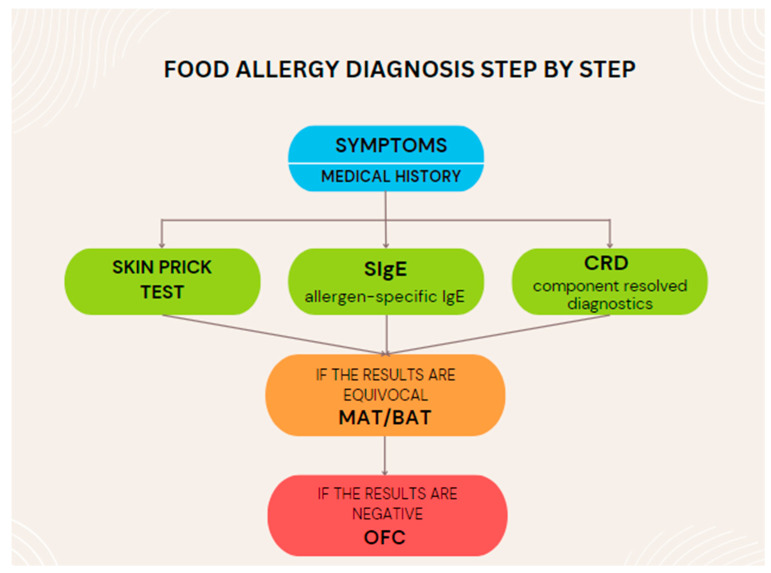
Food allergy diagnostic summary. SIgE—allergen-specific IgE; CRD—component resolved diagnostics; MAT—mast cell activation test; BAT—basophil activation test; OFC—oral food challenge test.

The diagnostic scheme includes an interview, physical examination, and a SigE test (CRD may be considered) and/or SPT (a positive SPT may indicate that the body is allergic to a given allergen but does not confirm it). These tests have high sensitivity but low specificity. Their implementation does not involve high costs; they are safe and used routinely. Performing the OFC test (or BAT/MAT when available) is recommended when the above-mentioned tests are negative despite a positive interview, or when the tests are positive but the interview is inconclusive [46]. OFC is associated with the risk of an acute allergic reaction of unpredictable severity. The test is time-consuming, but due to its specificity, it is still considered the gold standard, despite the development of molecular techniques and the scientific community’s opinion to abandon its use [59,61]. A summary of the diagnosis of FA is presented in Figure 3.

### 3.4. Treatment

In recent years, there has been significant progress in the treatment of food allergies. The GA2LEN Task Force suggests that patients with a documented food allergy should avoid the offending food unless their individual circumstances and risks allow for some consumption, as advised by their healthcare professionals [26].

The first form of therapy is oral immunotherapy (OIT). It involves the gradual administration of a food allergen orally under supervision, starting with minimal doses and then gradually increasing them until a maintenance dose is reached at an interval of 6 months [62]. Despite the effectiveness of OIT in desensitization, daily consumption of allergenic foods is stressful and is also burdened with dose-dependent side effects, which makes further compliance difficult. Gastrointestinal symptoms and severe anaphylaxis may occur. Attempts are underway to add monoclonal antibodies against IgE to reduce the severity of the side effects. The durability of such immunotherapy is also questionable, as food allergies usually recur within a few months or weeks [60,62].

Another form is epidermal immunotherapy (EPIT), which uses a system of transepidermal patches to continuously and non-invasively administer a food allergen through intact skin. Sweating of the skin dissolves the allergen and distributes it in the stratum corneum of the epidermis [49]. The use of EPIT increases the threshold dose of the food allergen. The side effects are local and milder compared to OIT [60,62].

An alternative treatment is sublingual immunotherapy (SLIT). It involves the daily administration of an allergen extract to the sublingual space of the patient’s mouth (the extract is placed under the tongue for 2–3 min and then swallowed). SLIT is associated with increased tolerance to the allergen, which is captured by the dendritic cells of the mucosa. The therapy is well tolerated, and side effects are minimal (itching or tingling in the mouth and throat). However, the effectiveness of this type of desensitization is low [49,63].

Omalizumab (OMA, a monoclonal antibody against IgE) is the best-studied biological drug that is used during multi-allergen oral immunotherapy, which reduces the likelihood of a severe allergic reaction during the test. The use of omalizumab in monotherapy also has benefits: OMA has an immunomodulatory effect (among others, it activates Treg lymphocytes, reduces the production of IgE, and induces the production of IgG4) and increases the tolerated dose of the allergen. Meta-analyses suggest that the use of OMA improves the quality of life because it reduces the need for an allergen-limiting diet, decreases the risk of allergic reactions from accidental contact with food, and minimizes the risk of anaphylaxis. Despite the promising results of previous meta-analyses, there is still a need for further research on the use of OMA as monotherapy or in combination with OIT. It is necessary to establish clear guidelines regarding the duration of therapy and the dosage [50,64].

Ligelizumab is a new-generation monoclonal antibody with 88-fold higher binding IgE compared to omalizumab [65]. Its subcutaneous use as monotherapy is currently being investigated in people allergic to peanuts [60].

With the progress of food allergy research, the impact of intestinal microbiome dysbiosis has been demonstrated; therefore, probiotics are considered in therapies to introduce new species into the intestines or manipulate existing ones. The immune-modulating and allergy-protective effects of the gut microbiota are thought to be mediated by metabolites produced from the fermentation of dietary fiber. These metabolites, such as SCFAs, regulate the size and function of the colonic Treg pool [66]. A beneficial effect of complementary therapy with probiotics containing strains of *Lactobacillus rhamnosus* GG, Bifidobacterium bifidum, and mixtures of many species is indicated, and further research is ongoing [60]. Probiotics have a positive effect on the intestinal epithelial barrier, which limits the entry of allergens into the blood. They modulate the immune system by attaching to endothelial cells, producing antibacterial substances, competing with pathogenic microorganisms, and acidifying the intestinal environment [67]. The rationale for the use of probiotics in food allergies is grounded in their potential to reduce inflammation while maintaining a favorable safety profile. In the treatment of peanut allergy, the combination of recombinant allergens with certain strains of probiotics has been shown to be a safe and immunologically effective strategy, given the modulating properties of probiotics in the immune system. Probiotic supplementation is carried with the goal of improving peanut desensitization and oral tolerance. Scientists have effectively used *Lactobacillus rhamnosus* CGMCC and *Lactobacillus rhamnosus* ATCC 53103 in peanut allergy therapy [68]. While results from some of the trials suggest that probiotics may be useful for food allergies, the most recent Cochrane review on this topic concluded that the data are insufficient thus far to recommend probiotic supplementation for food allergies. Statements from the World Allergy Organization comment on the limited and “very low-quality evidence” on this topic [66]. Moreover, the GA2LEN Task Force makes no recommendation for or against any prebiotics, probiotics, or synbiotics that have been evaluated so far for managing food allergies [26]. Prebiotics are nutrients that are not digested and promote the growth and activity of bacteria in the intestinal microbiome. The most commonly used prebiotics are indigestible carbohydrates, such as fructooligosaccharides (FOS) and galactooligosaccharides (GOS). Also, fiber is a prebiotic that many gut bacteria use as a nutrient, which in turn produces short-chain fatty acids, which are thought to beneficially inhibit allergic inflammation [69]. There are many benefits of administering prebiotics, including modulating intestinal function and strengthening the intestinal barrier, which limits the translocation of allergenic proteins into the bloodstream [68].

### 3.5. Quality of Life

Patients suffering from food allergies may experience a significant deterioration in their quality of life compared to the period before diagnosis and symptoms (health-related quality of life—HRQL). Quality of life refers to an individual’s subjective perception of their position in life, which is influenced by their living conditions, personal experiences, and values. Each of these dimensions is influenced by factors such as the environment, socio-economic status, sense of security, and physical, mental, social, and emotional health [70].

Patients are afraid of severe allergic reactions after eating food, and therefore, death. Anaphylaxis is unpredictable, and a patient’s health condition can change from fully functional to life-threatening in a matter of minutes. However, meta-analyses indicate that the number of deaths caused by food allergies is lower than the population risk of sudden death from another cause. The trick is to maintain a balance of reactions in the patient; it is important to be appropriately vigilant about the occurrence of symptoms and at the same time not to fall into excessive anxiety, which makes the ubiquity of food in everyday life difficult. Carrying an epinephrine auto-injection solution (epipen) can increase feelings of safety and comfort and reduce anxiety, but it can also cause anxiety as patients believe that the food allergy is so severe that adrenaline may be needed [47,71,72].

Moreover, constant allergen avoidance and exclusion diets are burdensome for patients and their families. The problem is reading product labels in stores and cooking meals carefully, and the greatest stress may be visits to restaurants. Food producers often abuse the notes “may contain eggs, nuts, etc.” to avoid responsibility for allergic reactions in consumers, which causes them to give up many food products and generates stress [70,72].

Avoiding certain foods may affect the nutritional status of adult patients. People with food allergies follow a restrictive diet and may not receive enough essential nutrients, which negatively affects their overall health. Patients who avoid dairy products are particularly at risk because of lower calcium levels in their bodies, which results in bone loss and impaired skeletal muscle function. A gluten-free diet for people allergic to wheat may cause vitamin deficiency, especially folic acid. Limiting the consumption of nuts and legumes results in reduced levels of omega-3 fatty acids, proteins, and soluble fibers. Therefore, doctors should pay special attention to the nutritional status of their patients [73].

Young adults may also experience bullying and social isolation due to food allergies. They limit their social life and do not participate in going out or in parties. Some scientists believe that this may be due to the need to change the lifestyle of people with food allergies, as they must exclude certain foods, which is not easy when they are with peers in canteens, pubs, and restaurants. Moreover, the need to carry emergency aid in the form of an epipen increases the anxiety associated with being in public places. People with food allergies experience ridicule, have food waved in their faces, or are forced to eat an allergenic meal, which may result in fear of contact with people and even depression [74].

Research indicates that adults with food allergies experience higher healthcare costs than controls, which is associated with an increased number of doctor visits per year (10/11 visits per year vs. approximately 6/7 visits in control groups). The cost difference was approximately USD 927, and similar results were obtained in each country. This places a significant burden on the budget of sick people and their families [71,75].

**Figure 4:**
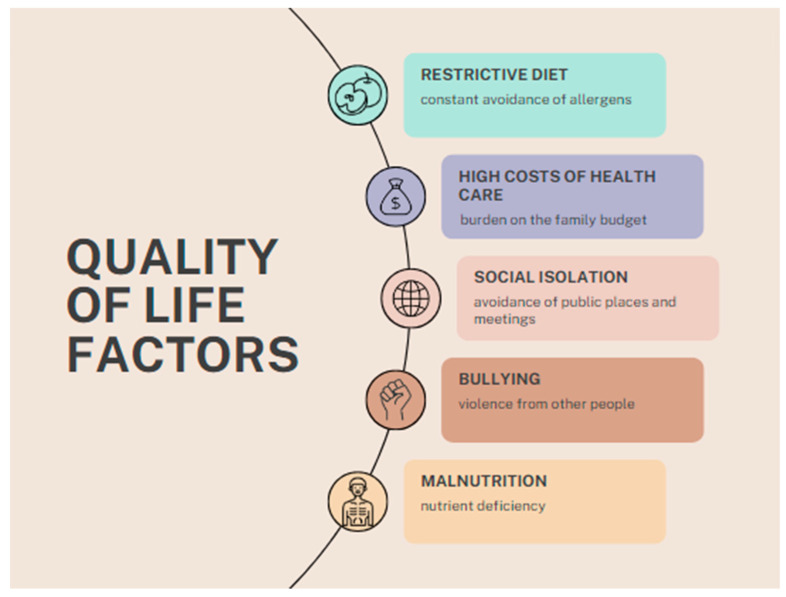
Summary of quality of life factors.

A recent study identified the challenges posed by the COVID-19 pandemic for people with food allergies. On the one hand, families ate meals at home, which increased their sense of security and control over food intake. On the other hand, shopping was problematic (especially at the beginning of the pandemic), and stores lacked basic allergen-free foodstuffs due to other people stockpiling food. However, allergy treatment and diagnosis were delayed because they were classified as elective procedures that could be postponed [71]. A summary of the main quality of life factors is presented in Figure 4.

## 4. Conclusions

This paper summarizes and systematizes the latest knowledge on food allergies in children and adults. The recent data suggest that food allergies affect a growing number of infants, children, and adults around the world. Its pathogenesis consists of many complex mechanisms, and various factors have a major impact on the occurrence of FA. There are many manifestations of food allergies, and some of them depend on the patient’s age. The main difference between children and adults is the allergens that cause the disease (cow’s milk versus shellfish). Due to the constantly increasing number of allergy cases, the need for improvements in diagnosis, prevention, and treatment remains pressing and important. New therapeutic methods are promising, such as the use of monoclonal antibodies and immunotherapy. However, there is still a need to find a diagnostic method other than the gold standard, OFC, which may have complications.

## Data Availability

Data sharing is not applicable as no datasets were generated or analyzed during the current study.
